# The Role of BANK1 in B Cell Signaling and Disease

**DOI:** 10.3390/cells10051184

**Published:** 2021-05-12

**Authors:** Gonzalo Gómez Hernández, María Morell, Marta E. Alarcón-Riquelme

**Affiliations:** 1 GENYO, Center for Genomics and Oncological Research, Pfizer, University of Granada, Andalusian Government, PTS, 18016 Granada, Spain; gonzalo.gomez@genyo.es (G.G.H.); maria.morell@genyo.es (M.M.); 2 Department of Environmental Medicine, Karolinska Institutet, 17167 Solna, Sweden

**Keywords:** B cells, B cell receptor, CD40, autoimmunity, genetic association, B cell scaffold with ankyrin repeats, TLR7, BLK, LYN, TRAF6

## Abstract

The B cell scaffold protein with ankyrin repeats (BANK1) is expressed primarily in B cells and with multiple but discrete roles in B cell signaling, including B cell receptor signaling, CD40-related signaling, and Toll-like receptor (TLR) signaling. The gene for BANK1, located in chromosome 4, has been found to contain genetic variants that are associated with several autoimmune diseases and also other complex phenotypes, in particular, with systemic lupus erythematosus. Common genetic variants are associated with changes in BANK1 expression in B cells, while rare variants modify their capacity to bind efferent effectors during signaling. A BANK1-deficient model has shown the importance of BANK1 during TLR7 and TLR9 signaling and has confirmed its role in the disease. Still, much needs to be done to fully understand the function of BANK1, but the main conclusion is that it may be the link between different signaling functions within the B cells and they may act to synergize the various pathways within a cell. With this review, we hope to enhance the interest in this molecule.

## 1. Introduction

The importance of the gene B cell scaffold with ankyrin repeats (BANK1) was gained due to the genetic studies on systemic lupus erythematosus and other diseases that followed. The role of BANK1 in B cell signaling had been somewhat difficult to define; however, since the commencement of mouse studies, its role in the disease and TLR7 signaling has become evident.

BANK1 was first identified by Yokoyama et al. [1] when using a solid-phase phosphorylation screening in the search for Lyn substrates. A novel ankyrin repeat-containing protein was identified and sequenced. Based on the sequence information, the coding sequence of the protein was obtained by RT-PCR of RNA isolated from a Daudi cell line. In general, the major isoform of BANK1 of 755 amino acids was identified, with a molecular weight of 85,500 Da.

The initial gene expression analyses using northern blots and mouse tissues found that BANK1 was expressed in the spleen and lymph nodes of adult mice, but not in Rag-/- or SCID mice. When looking into the B cell development populations, BANK1 was observed in immature and recirculating B cells, but not in pro-B or pre-B cells. Looking into separated splenic B cells, BANK1 was expressed in IgM^hi^IgD^−^, IgM^hi^IgD^hi^ and the most mature IgM^lo^IgD^hi^ populations. The authors suggested that the expression of BANK1 depended on the expression of the B cell receptor (BCR); however, there is evidence showing that BANK1 is also expressed in dendritic cells (BioGPS).

It was also found that BANK1 had some homology with other ankyrin-containing proteins, such as BCAP and the Drosophila protein Dof, although the overall homology with BCAP is 33%. The ankyrin repeats are located in amino acids 309-372 of BANK1, while a coiled-coil sequence of BANK1 is located in amino acids 647–675.

Thus, BANK1 was now entering the scientific publication sphere, but more was to come.

## 2. The Genetic Associations of Variants of BANK1 with Diseases

Table 1 shows the main genetic associations of BANK1 polymorphisms and the diseases where these associations have been found. A genome-wide association study identified *BANK1* as a susceptibility gene for systemic lupus erythematosus (SLE) [2]. This study identified the D2 isoform of BANK1 by RT-PCR. It was observed that two closely linked single nucleotide polymorphisms (SNPs) were strongly associated in a European population, the non-synonymous variant rs10516487 located in the coding exon 2 and causing an R61H change in the protein, and rs17266594 located in the preceding intron 1 in a branch-point site. A third variant, rs3733197, creates an A383T change in the ankyrin domain and was weakly associated. The SNP association was also associated with a differential expression of the D2 isoform, whose expression was increased in homozygous for the protective allele of rs17266594. Initially, it was considered that the exonic variant could affect the binding of the inositol-3-phosphate receptor by BANK1; however, the role of BANK1 in TLR7 signaling appears to be more relevant. Another important feature of the genetic association of the gene *BANK1* with SLE is that it was the major allele of rs10516487 that was showing the association, R61 being the major allele and, hence, the risk variant. This genetic association was fully replicated by Guo et al. [3], a study that also showed an association with hematological and immunological SLE classification criteria. Further studies also replicated the association in Finns [4], and other independent studies in European populations [5,6,7,8]. In one of these studies using a cohort of over 7000 SLE cases and nearly 16,000 controls, the major risk variant identified was rs10028805 [7], later confirmed in African Americans [9]. Further studies on the genetic association of *BANK1* with SLE showed an association with the presence of anti-dsDNA autoantibodies [10]. The genetic association of *BANK1* polymorphisms with SLE was also confirmed in Chinese, Hong Kong Chinese, and Thai populations [11,12,13]. However, *BANK1* has been weakly associated in Mexican populations [14] or not at all in a population enriched for Amerindian ancestry [15].

Of importance is the genetic association of *BANK1* in African Americans. The genetic association is particularly strong [16,17], and two independent effects have been associated with *BANK1* in SLE patients with AA ancestry [11].

Further association studies were performed with other diseases, for example, primary antiphospholipid syndrome, where the variants of *BANK1* were not associated [25], or rheumatoid arthritis (RA), where an association was observed [22] but was not replicated in a second study [18]. Similarly, *BANK1* genetic polymorphisms were associated with systemic sclerosis [26] and replicated in a second European population [21], particularly in diffuse SSC. A more recent study identified, using whole exome sequencing [18], potential causal variants for diffuse systemic sclerosis in patients who also had interstitial lung disease [27]. Regarding cancers, a genetic association of *BANK1* SNP rs10028805 with chronic lymphocytic leukemia has been reported [23]. Finally, a study reported the well-replicated association of *BANK1* with serum LDL cholesterol levels in Koreans, represented by the association with the ankyrin non-synonymous SNP rs3733197 [24]. Of interest, in this regard, a study tested up to 62 GWAS loci from a GWAS, investigating the association of body mass index [26] with functional knock-out screens in *Drosophila melanogaster* brain and fat tissue [28]. While the genetic association pointed to *SLC39A8*, the closest to the associated SNP rs13107325, *BANK1* was one of the sets of genes functionally corroborated.

A genetic association has also been observed with primary Sjogren’s syndrome (pSS) [29]. This study analyzed salivary gland biopsies associating the allele frequencies with patients having ectopic germinal center structures and found a positive association with *BANK1* polymorphisms as well as other genes, such as *AICDA* and *BCL2*. Association between variants of *BANK1* and Sjogren’s syndrome was not replicated in a Chinese population [30] or any major GWAS of the disease [31], while BLK was indeed associated [30,31]; however, no study has analyzed the potential genetic role of *BANK1* in the development of lymphoma in pSS or RA.

Several studies analyzed the genetic interaction between *BANK1* and a second SLE-associated B cell gene, *BLK* [32,33], and one of the studies, as mentioned in the previous section, showed the physical interaction between the proteins of both genes [33]. Such interaction has been identified in several populations [19] but also in several diseases, apart from SLE, such as SSC [32] and RA [34]. One study that also replicated the interaction in a Chinese population showed that the relative expression level of mRNA for *BLK* was lower in presence of the risk allele rs2736340, and a significant linear association of relative mRNA expression of *BLK* and *BANK1* was also detected as indicative of this interaction in SLE patients [35]. Finally, a study analyzing a non-synonymous mutation in *BLK* (A71T) was related with decreased levels of BLK protein and binding to BANK1 through the impairment of the function of the SH3 domain of BLK, confirming once again the existence of the interaction at the protein level [36].

**Figure 1:**
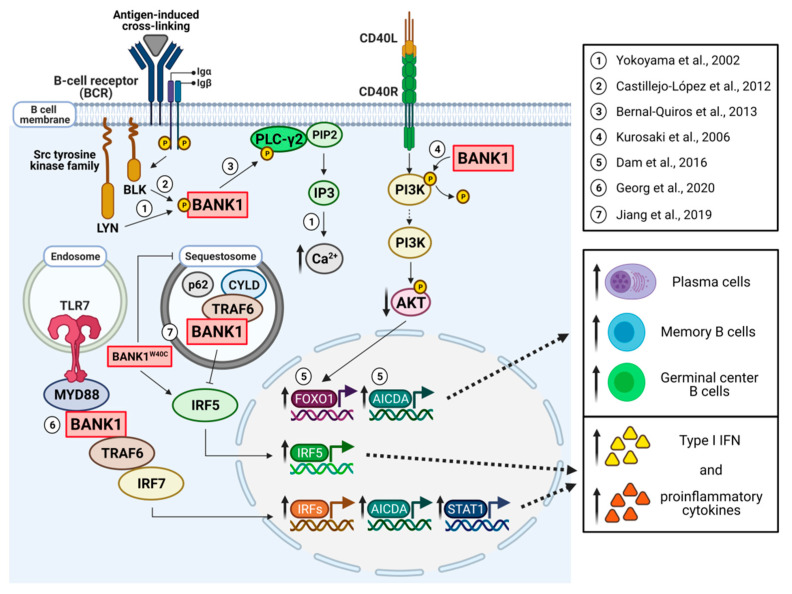
Model of the role of BANK1 in B cell signaling. BANK1 is primarily expressed in B cells and is involved in B cell signaling pathways. During BCR activation, BANK1 becomes tyrosine phosphorylated and binds the Src family kinases LYN and BLK (1-2) [1]. BANK1 also interacts with PLC-γ2 [44], regulating BCR-induced calcium mobilization. BANK1 attenuates CD40-mediated AKT activation (4) [45]. Following the PI3K/AKT pathway, the transcription factors FOXO1 and AICDA appear to increase in subjects with BANK1 risk variants, resulting in an increase in plasma cells, memory B cells, and germinal center B cells (5) [38]. BANK1 interacts with TRAF6 and MyD88 through endosomal TLR7 activation (6), triggering an IRF7-dependent production of IFNα and several proinflammatory cytokines. In the absence of exon 2, in BANK1–D2, there is a significant decrease in binding to MyD88 compared to BANK1–FL [37]. Finally, BANK1 binds to TRAF6, forming a complex with the sequestosome protein p62 and the deubiquitinating enzyme CYLD. In these structures, BANK1 promotes TRAF6 sequestration, diminishing IRF5 activation and type I IFN induction, and this is modified by a mutation in the amino acid position 40 in exon 2 (W40C) (7) [20].

One study analyzed a family with multiple cases of SLE and found rare coding variants in both genes *BANK1* and *BLK* that segregated in families [20]. The *BANK1* variant was a W to C change in amino acid position 40 located in exon 2 (W40C). Importantly, this study found that the expression of *BANK1* 40C in HEK293T cells led to reduced formation of the so-called sequestosomes, and, therefore, when expressed with TRAF6 and IRF5, BANK1 40C could not repress TRAF6-mediated IRF5 nuclear localization, leading to increased IRF5 activation and induction of type I interferon, important in the pathogenesis of SLE (Figure 1). In a second study, using immunoprecipitation experiments, Georg et al. showed that the same change (40C) promoted the binding of BANK1 to MYD88 [37] and led to increased production of IL-8 in a transfection system (Figure 1). Both studies show the importance of BANK1 in mediating TRAF6 activity through several mechanisms and that the 40C variant is indeed a risk mutation, promoting the development of SLE in families.

Another study went deeper into the role of the BANK1 gene in alterations in peripheral B cell signaling [38]. This study found that B cells of *BANK1* risk allele carriers of rs10516487 had increased the basal expression of the FOXO1 protein and increased the expression of *FOXO1* target genes upon stimulation with anti-IgM and CD40. FOXO1 is an important transcriptional target of the PI3K/AKT pathway, and, as described previously, it is modulated in the absence of *BANK1* (Figure 1). Activation of the PI3K/AKT pathway was severely decreased in individuals who were carriers of the risk alleles of *BANK1*. This was observed in reduced phosphorylation of AKT and PLCg2, showing a general reduction in BCR signaling. Of interest, upon BCR/CD40 stimulation, BANK1 full-length isoform expression was increased in risk allele carrier B cells and correlated strongly with mRNA levels of AICDA and SELL (CD62L) as well as FOXO1 (Figure 1). This is interesting, as AICDA is involved in class switching, while SELL or CD62L, on the other hand, is involved in B cell homing into the lymph nodes.

Of interest, BANK1 has also been found to be genetically linked to SLE in dogs, particularly with the presence of antinuclear antibodies and with similar gene expression patterns as found in the human disease [39].

## 3. The Role of BANK1 in B Cell Signaling

The BANK1 protein is constituted by three conserved domains: two double ankyrin repeat-like [12] motifs, a coiled-coil (CC) domain, and a Dof/BCAP/BANK (DBB) motif [40]. BANK1 also includes in its sequence several tyrosine- and proline-rich regions that could provide docking sites for SH2- and SH3-containing proteins. At the protein level, four isoforms have been reported, of which two have been the subject of several studies. The first isoform contains the canonical sequence (785 amino acids), is generated by alternative splicing, and is named the full-length isoform (FL). The other isoform of interest, named Delta2 (D2) (652 amino acids), lacks exon 2, which encodes for a putative conformational TIR domain [41]. Increased expression of this isoform and lower expression of the FL isoform have been linked, genetically, to protection against SLE [2]. It is unclear how splicing of the isoforms of BANK1 occurs and how the D2 is formed. One work suggests that the non-synonymous variant rs10516487 in exon 2 does influence splicing efficiency through the creation of an exonic splicing enhancer site that binds the SRp40 splicing factor [42]. Furthermore, the FL isoform containing the risk variant (R61) is capable of forming larger scaffold complexes in the cell cytoplasm, that is, has increased potential of multimerization. A functional TRAF6 binding motif has been reported as well as the confirmation of the presence of a functional TIR domain [37].

BANK1 is mainly expressed in B cells and, as a scaffold, it is involved in B cell signaling pathways. As mentioned previously, the first study demonstrated that BANK1 binds to the Scr tyrosine kinase LYN [1] (Figure 1). In this line, posterior studies showed that BANK1 also binds to BLK when tested in a B cell line and primary and naive B cells.

BANK1 also interacts with phospholipase 2C (PLCg2) (Figure 1). PLCg2 is part of a major group of signaling switch molecules involved in the formation of the second messenger inositol 1,4-5-triphosphate (IP3) and diacylglycerol. PLCg1 and PLCg2 have two Src homology domains (SH2) and an SH3 domain. PLCg2 is the one expressed in hematopoietic cells and is important in the regulation of immune activation [43]. This interaction of BANK1 and PLCg2 is promoted by engagement to the BCR and through binding with the proline-rich motifs and the phosphorylation of tyrosine residues on BANK1. The formation of the BANK1–PLCg2 complex is modulated by its sub-cellular location and the kinase activity of BLK, suggesting a role of BANK1 in modulating BCR signaling through a BANK1–BLK interaction [44].

The first study using the mouse deficient of BANK1 (*BANK1^−/−^*) [45] showed that that the IgM B cell responses to T-dependent antigens, numbers of mature B cells, spontaneously formed germinal centers, and levels of IgG2a were enhanced in these mice, a phenotype blocked by a double knock-out of *BANK1* and *CD40*. In vitro, CD40-mediated proliferation and cell survival were increased in *BANK1^−/−^* mice accompanied by enhanced *Akt* activation [45].

BANK1 has a role in type I interferon signaling and cytokine production. BANK1 interacts with TRAF6 and MyD88 as demonstrated with immunoprecipitation studies [37] (Figure 1). TRAF6 forms a complex with MyD88 and IRF7 after TLR7, TLR8, or TLR9 stimulation, triggering the production of IFNalpha [46]. In in vitro studies using the HEK293 cell line, the absence of exon 2, the BANK1–D2 isoform lacking the TIR domain, exhibited a significant decrease in binding to MyD88 compared to BANK1–FL [37], demonstrating that the putative TIR domain of BANK1 is indeed functional. As mentioned above, this study identified TRAF6-binding motifs confirmed using point mutations and decoy peptide experiments. These experiments showed the requirement to form the complex through the C-terminal domain with TRAF6 and the TIR domain for MyD88. Polyubiquitination of BANK1 is mediated by lysing 63 (K63) through the TIR domain. This ubiquitination is important for the activation of the signaling pathway and cytokine production, as shown [37]. In the same cell line, others have demonstrated that BANK1 binds to TRAF6 forming a complex with the sequestosome protein p62 and with the deubiquitinating enzyme CYLD [20], which plays a critical role in regulating TLR signaling [47]. The authors proposed that BANK1 promotes TRAF6 sequestration diminishing IRF5 activation and IFN induction. The above results need to be further explored to better understand the BANK1 function in the various signaling pathways. 

## 4. Mechanistic Aspects of the Role of BANK1 in Experimental Disease Model Systems

To date, very little is known about the role that BANK1 may play in different diseases. Following the report of the genetic association of BANK1 with SLE [2] and RA [22], a study discovered that BANK1 had a role in islet primary nonfunction (PNF), a serious problem in islet transplantation [48]. The transplantation of isolated islets from a donor pancreas is used to treat type 1 diabetes mellitus, an autoimmune disease characterized by selective destruction of pancreatic beta cells causing a gradual deficiency of insulin. This study investigated if DcR3, a TNF family receptor, could protect islets from apoptosis, as it may occur through the FasL, LIGHT, or TL1A pathways. By generating transgenic mice expressing human DcR3, two molecules, *Adcyap1* and *BANK1*, were found to reduce β cell apoptosis by modulating their expression. With gene expression analysis, it was shown that overexpression of *Adcyap1* or reduced expression of *BANK1* in transgenic DcR3 mice prevented cells from undergoing cytokine-triggered apoptosis, suggesting the existence of a novel mechanism of islet protection and survival. There is evidence that IL-1β- and glucose-induced β cell apoptosis is calcium flux dependent [49]. Hence, a possible mechanism for DcR3 to protect β cells is to inhibit cytokine-induced *BANK1* up-regulation, which in turn, prevents calcium mobilization, as previously described for *BANK1* in B cells [1]. It must, however, be mentioned that the changes in calcium mobilization dependent on *BANK1* have not been confirmed by any other study.

As mentioned above, BANK1 has a role in B cell signaling. During BCR activation, BANK1 becomes tyrosine phosphorylated and is able to bind the Src family kinases Lyn and Blk, acting as an adaptor or scaffold protein connecting these protein tyrosine kinases (PTKs) to IP_3_R receptors, which are in the same family as the B cell adapter BCAP [1,41]. In addition, it has been shown that exon 2 of human *BANK1* encodes an N-terminal toll/IL-1 receptor [26] domain that is shared by BCAP. Toll-like receptors (TLRs) use adapters that contain the TIR domain, such as MyD88 and TRIF, to induce activation of transcription factors, including NF-κB, MAP kinases, and IFN regulatory factors [41]. In this regard, TLR9 is one of the most relevant endosomal TLRs in B cells, and TLR9 signaling is believed to have a critical role in autoimmunity [50]. Because of the role of BANK1 as a TIR-containing adaptor and the role of TLR9 in autoimmunity, the effect of BANK1 in TLR9 signaling was studied. Using *BANK1* deficient mice and stimulation with the TLR9 agonist CpG, a reduction in MAPK p38 phosphorylation was observed in purified splenic B cells as compared with littermate control wild-type mice. *BANK1* deficiency also led to the reduced production of the proinflammatory cytokine IL-6 in response to CpG alone or in combination with BCR ligation. However, there was no reduction in IL-6 mRNA, so an alternative mechanism had to be found. It was then found that *BANK1* deficiency also reduced CpG-induced MNK1/2 and eIF4E phosphorylation kinases, which form the MNK1/2/eIF4E/eIF4G pathway of the translation initiation process, controlled by p38 [51], thus reducing translation of IL-6. 

Subsequent studies were aimed at investigating the effects of BANK1 in the context of an autoimmune disease in a lupus mouse model. In order to do so, crosses of B6.*Sle1*.*yaa* with *BANK1*^−/−^ mice were generated [52]. This model carries the *Sle1* locus, which is analogous to a chromosomal region linked with SLE susceptibility in humans [53], and the *yaa* locus, known to be the result of the translocation of an X chromosome region to the Y chromosome resulting in a duplication of a large number of genes, including *Tlr7* [54], another endosomal TLR receptor, which contains TIR domains and is associated with autoimmune pathogenesis [55]. Increased expression of TLR7 is enough to induce a lupus-like disease [56]. In this study [52], the effect of *BANK1* deficiency on major lupus phenotypes was observed, among which were a reduction in mortality and in the production of total IgG and IgG anti-dsDNA antibodies, as well as the pathogenic isoform IgG2c, in B6.*Sle1*.*yaa*.*BANK1^−/−^* mice compared with B6.*Sle1*.*yaa*.*BANK1^+/+^*. The proinflammatory cytokine IL-6 was also reduced in the absence of *BANK1* as previously seen [51]. By flow cytometry analysis, it was observed that *BANK1* deficiency restores the cellular phenotypes of splenic lymphocytes and myeloid cells. The only effect observed in T cells was the normalization of the expression of CXCR4 on follicular T helper cells (T_fh_), suggesting that *BANK1* has effects on the formation of extrafollicular foci. In addition, in vitro experiments stimulating TLR7 and TLR8 agonists with imiquimod and resiquimod, respectively, showed that *BANK1* regulates TLR7-induced signaling pathways in B cells, leading to the reduced expression of *Aicda*, as well as interferon response factor genes (*Irf1*, *Irf7* and *Irf9*), *Stat1*, and type I IFN genes (*Ifna4* and *Ifnb*) in B6.*Sle1*.*yaa*.*BANK1*^−/−^ mice [52]. 

BANK1 has been implicated in B cell lymphomagenesis, a tumorigenesis of immature B cells [57]. The SL/KH mouse model, a model of spontaneous pre-B-lymphomas, was used. By qRT–PCR, it was observed that overexpression of *Zfp521*, a putative gene involved in the induction of B cell lymphomagenesis, caused a marked increase in the expression of pre-BCR-related genes, including *BANK1*. Knockdown of *BANK1* reduced the proliferation of pre-B cells.

BANK1 has also been related with colitis, an inflammatory disease also known as Crohn’s disease. Employing a novel experimental procedure, based on the immuno-capture of MHCII complexes obtained from lymph nodes followed by a mass spectrometric analysis of eluted peptides, peptides derived from a subset of proteins, including BANK1, were identified in a dextran sodium sulfate (DSS)-induced colitis mouse model [58].

Finally, a study using collagen-induced arthritis (CIA) mice, the classic murine model of rheumatoid arthritis (RA), observed a reduction in *BANK1* levels in the spleen, peripheral blood, and lymph nodes of CIA mice during the acute stage of arthritis, which had a negative correlation with disease severity and autoantibody production [59].

## 5. Conclusions

There is still much to be done to understand the role of BANK1 in B cell signaling. Moreover, some evidence suggests that BANK1 may also be expressed in myeloid cells and plasmacytoid dendritic cells, but no work has been performed on these aspects. Being a scaffold important in the signal transduction of various signaling pathways, how these pathways may converge or synergize through BANK1 is still completely unknown. *BANK1* holds major risk alleles for several related antibody-mediated autoimmune conditions, and, thus, it warrants much greater attention.

## Figures and Tables

**Table 1:** List of genetic associations of polymorphisms of BANK1.

Disease	SNPs ^1^	Location in Protein	Study
SLE	rs10516487	TIR domain	[2]
SLE	rs17266594	--	[2]
SLE	rs3733197	Ankyrin repeats	[2]
SLE	rs17266594	---	[3]
SLE	rs10028805	---	[4,5,6,7,8,9,12,13,14,16,17,18,19]
SLE	W40C	TIR domain	[20]
Diffuse SSC	rs10516487	TIR domain	[21]
Diffuse SSC	rs3733197	Ankyrin repeats	[21]
RA	rs10516487	TIR domain	[18,22]
Chronic Lymphocytic Leukemia	rs10028805	---	[23]
LDL Cholesterol	rs3733197	Ankyrin repeats	[24]

^1^ Single nucleotide polymorphisms associated in the study.

## Data Availability

No new data were created or analyzed in this study. Data sharing is not applicable to this article.
